# Sapap3 deletion causes dynamic synaptic density abnormalities: a longitudinal [^11^C]UCB-J PET study in a model of obsessive–compulsive disorder-like behaviour

**DOI:** 10.1186/s13550-020-00721-2

**Published:** 2020-11-13

**Authors:** Dorien Glorie, Jeroen Verhaeghe, Alan Miranda, Stef De Lombaerde, Sigrid Stroobants, Steven Staelens

**Affiliations:** 1 Molecular Imaging Center Antwerp (MICA), University of Antwerp, Universiteitsplein 1, Wilrijk, Belgium; 2 Department of Nuclear Medicine, Antwerp University Hospital, Edegem, Belgium

**Keywords:** Obsessive–compulsive disorder (OCD), SAP90/PSD-95-associated protein 3 (Sapap3), Positron emission tomography (PET), Synaptic vesicle protein 2A (SV2A), [^11^C]UCB-J, [^3^H]UCB-J, Autoradiography, Grooming

## Abstract

**Background:**

Currently, the evidence on synaptic abnormalities in neuropsychiatric disorders—including obsessive–compulsive disorder (OCD)—is emerging. The newly established positron emission tomography (PET) ligand ((R)-1-((3-((11)C-methyl-(11)C)pyridin-4-yl)methyl)-4-(3,4,5-trifluorophenyl)pyrrolidin-2-one) ([^11^C]UCB-J) provides the opportunity to visualize synaptic density changes in vivo, by targeting the synaptic vesicle protein 2A (SV2A). Here, we aim to evaluate such alterations in the brain of the SAP90/PSD-95-associated protein 3 (Sapap3) knockout (ko) mouse model, showing an abnormal corticostriatal neurotransmission resulting in OCD-like behaviour.

**Methods:**

Longitudinal [^11^C]UCB-J µPET/CT scans were acquired in Sapap3 ko and wildtype (wt) control mice (*n* = 9/group) to study SV2A availability. Based on the Logan reference method, we calculated the volume of distribution (*V*_T(IDIF)_) for [^11^C]UCB-J. Both cross-sectional (wt vs. ko) and longitudinal (3 vs. 9 months) volume-of-interest-based statistical analysis and voxel-based statistical parametric mapping were performed. Both [^11^C]UCB-J ex vivo autoradiography and [^3^H]UCB-J in vitro autoradiography were used for the validation of the µPET data.

**Results:**

At the age of 3 months, Sapap3 ko mice are already characterized by a significantly lower SV2A availability compared to wt littermates (i.a. cortex − 12.69%, *p <* 0.01; striatum − 14.12%, *p <* 0.001, thalamus − 13.11%, *p <* 0.001, and hippocampus − 12.99%,* p* < 0.001). Healthy ageing in control mice was associated with a diffuse and significant (*p <* 0.001) decline throughout the brain, whereas in Sapap3 ko mice this decline was more confined to the corticostriatal level. A strong linear relationship (*p <* 0.0001) was established between the outcome parameters of [^11^C]UCB-J µPET and [^11^C]UCB-J ex vivo autoradiography, while such relationship was absent for [^3^H]UCB-J in vitro autoradiography.

**Conclusions:**

[^11^C]UCB-J PET is a potential marker for synaptic density deficits in the Sapap3 ko mouse model for OCD, parallel to disease progression. Our data suggest that [^11^C]UCB-J ex vivo autoradiography is a suitable proxy for [^11^C]UCB-J PET data in mice.

## Background

Obsessive–compulsive disorder (OCD) is a severe neuropsychiatric disease with a lifetime prevalence of 1–3% [1]. Patients are highly impaired in their daily functioning due to persistent intrusive thoughts (obsessions) and by engaging in time-consuming repetitive actions (compulsions) [2] to reduce the anxiety about their obsessions. The severity of this disorder is reflected by its ranking in the top ten causes of illness-related disability based on quality of life and lost earnings proposed by the World Health Organization [3]. Cortico-striato-thalamocortical (CSTC) circuit dysfunction was previously established as essential to OCD neurobiology [4–6] and mainly supported by neuroimaging studies [7]. However, OCD pathophysiology is complex and still little understood. This is likely attributed to the associated patient heterogeneity, the different comorbidities, and the medication history of OCD patients [8, 9]. Also, a considerable number of patients remain treatment refractory [10], which stresses the need to explore new targets, thereby working towards the development of novel and directed therapeutics. The barriers associated with clinical OCD research can be circumvented via the use of animal models. They provide the opportunity to further unravel and dissect the pathological mechanisms underlying compulsive(-like) behaviours [11–17].

A widely used model in preclinical OCD research is the SAP90/PSD-95-associated protein 3 (Sapap3; DLGAP3; GKAP3) knockout (ko) mouse model [11]. This protein is localized in the postsynaptic density (PSD) of excitatory synapses. The PSD is a multiprotein complex consisting of neurotransmitter receptors, scaffolding proteins, adhesion molecules, signalling enzymes, and cytoskeletal components [18, 19]. The spatial and the temporal organization of these components plays a central role in synaptic signalling and plasticity [20]. A key aggregate in this structure is the PSD-95/Sapap/Shank complex, which is crucial for synaptic development and transmission [21]. Mice lacking Sapap3 display synaptic dysfunction with aberrant neurotransmission at the level of (cortico)striatal synapses [11, 17, 22–26], resulting in a phenotype suggestive for OCD with excessive grooming and increased anxiety-like behaviour. Already at an early age this model shows a marked increase in grooming frequency, despite the absence of the typical skin lesions. In addition, an exacerbation of this behaviour was demonstrated, reflected by an important increase in grooming duration with ageing [27]. This is indicative of a dynamic pathological process in the Sapap3 ko mouse brain. On this basis, we can put forward in vivo longitudinal small-animal positron emission tomography (µPET) imaging as a suitable technique to quantify possible evolutions at the level of the synapse in this model. Recently, a novel biomarker for molecular imaging became available to visualize in vivo synaptic density changes. This new PET ligand ((R)-1-((3-((11)C-methyl-(11)C)pyridin-4-yl)methyl)-4-(3,4,5-trifluorophenyl)pyrrolidin-2-one) ([^11^C]UCB-J) [28, 29] targets the synaptic vesicle protein 2A (SV2A), which is an integral glycoprotein of the synaptic vesicle membrane and is omnipresent in virtually all synapses [30].[^11^C]UCB-J was previously shown to possess a high affinity and specificity for its target, with a rapid uptake and optimal kinetics in the mouse brain [31].

Also from a clinical point of view, there is emerging—mainly genetic—evidence for “synaptopathies” as a hallmark in the development of OCD [32–36] and other neuropsychiatric diseases [37–41]. Moreover, the SV2A targeting antiepileptic drug levetiracetam [42] was associated earlier with adverse psychiatric reactions, including the occurrence of obsessive–compulsive behaviours [43–45]. Differently, another case report demonstrated the ability of levetiracetam to treat a refractory OCD patient [46].

Given this emerging evidence together with previous findings in Sapap3 ko mice, we sought to investigate whether longitudinal aggravation of the OCD-like phenotype in Sapap3 ko mice is associated with in vivo changes in synaptic density via [^11^C]UCB-J µPET. Also, for validation purposes and as one of the first, we aim to perform ex vivo ^11^C autoradiography where multiple animals are injected with rapidly decaying [^11^C]UCB-J in tracer dose conditions [47] complementary to the traditional in vitro [^3^H] autoradiography where tissue slides are incubated with the tracer. This enables to investigate whether ex vivo [^11^C]UCB-J and in vitro [^3^H]UCB-J autoradiography can function as a proxy for the in vivo µPET measurements, with ex vivo [^11^C]UCB-J autoradiography better reflecting the physiological environment for tracer–protein interactions.

## Methods

### Mice

**Fig. 1:**
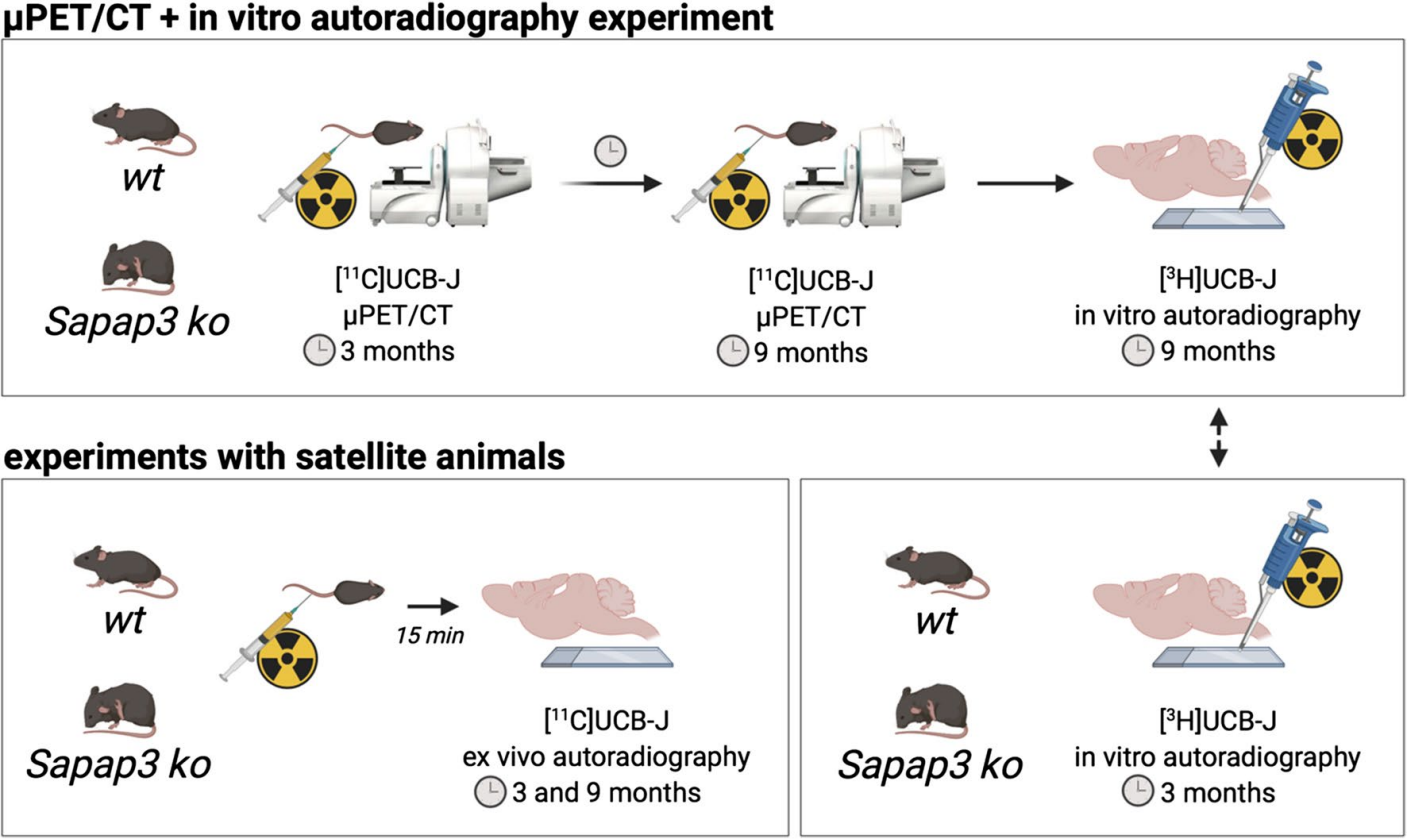
Overview of all the experimental procedures. (wt = wildtypes; ko = knockouts)

For the longitudinal µPET experiment, female Sapap3^−/−^ ko mice on a C57BL/6J background were bred in house at the University of Antwerp from heterozygous Sapap3^+/−^ breeder pairs (kindly obtained from Prof. Dr. G. Feng, Massachusetts Institute of Technology). Genotypes were determined via PCR of mouse ear DNA. All animals were scored biweekly for skin lesions, based on severity. The average age when skin lesions appeared in ko mice was 7.17 ± 1.61 mo. All Sapap3^−/−^ ko mice and age-matched wildtype (wt) C57BL/6J littermates (*n* = 9 per genotype) were co-housed in individually ventilated cages under controlled conditions (12-h normal light–dark cycles, 20–23 °C, and 50–55% relative humidity) with water and rodent food pellets ad libitum. Additional female satellite animals were kept under the same circumstances for [^11^C]UCB-J ex vivo (*n* = 3 per genotype and per age group) and for [^3^H]UCB-J in vitro (*n* = 5 per genotype for the 3-mo age group) autoradiography. Figure 1 provides an overview of all experimental procedures. These procedures were performed in accordance with the European Ethics Committee (decree 86/609/CEE). The study protocol was approved by the Animal Experimental Ethical Committee of the University of Antwerp, Antwerp, Belgium.

### Tracer synthesis

[^11^C]UCB-J synthesis was performed on an automated synthesis module (Carbosynthon I, Comecer, The Netherlands) adapting the previously described procedure to our system [29]. Briefly, the [^11^C]UCB-J compound was synthetized via a single-step carbon-11 labelling starting with a mixture of trifluoroborate and boric acid precursors (ratio 95:5), which was prepared prior to the synthesis and reacted with [^11^C]MeI already trapped into DMF. The reaction occurred in the presence of a Pd catalyst (Pd_2_(dba)_3_), P(o-tol)_3_, and a base (K_2_CO_3_) in DMF for 5 min at 100 °C. Prior to the addition of the mixture, trapping of [^11^C]MeI in the reactor with DMF was required for the reaction. The average radiochemical purity was greater than 99%.

The injected cold mass was aimed below 4.5 nmol/kg to avoid cold mass effects [47] (Table 1). For the µPET experiment, the mean molar activity (*A*_m_) at time of injection was 44.70 ± 7.99 MBq/nmol for the wt group and 44.85 ± 7.74 MBq/nmol for the ko group at the 3-mo timepoint. At 9 mo, the mean A_m_ was 40.20 ± 12.69 MBq/nmol for the wt group and 44.02 ± 12.96 MBq/nmol for the ko group.

**Table 1:** Overview of the animal and the scan parameters for the [^11^C]UCB-J dynamic µPET/CT scans with equal injected cold mass for both groups at both timepoints

Age	Genotype	Animal number	Body weight (g)	Injected dose (MBq)	Injected mass (nmol/kg)
3 mo	wt	8	21.29 ± 1.25	4.32 ± 0.74	4.56 ± 0.25
	ko	9	20.07 ± 1.15	4.18 ± 0.72	4.66 ± 0.18
9 mo	wt	6	25.80 ± 1.32	4.45 ± 1.43	4.29 ± 0.36
	ko	9	23.56 ± 1.56	4.54 ± 1.22	4.44 ± 0.21

Parameters are expressed as mean ± SD

mo = months; wt = wildtypes; ko = knockouts

### Longitudinal [^11^C]UCB-J dynamic µPET/CT scans

At the age of 3 and 9 mo all mice received a dynamic µPET/CT acquisition. Mice were anesthetized using isoflurane (IsoFlo®, Zoetis, USA) mixed with medical oxygen (induction 5%, maintenance 2%) and placed on a heated blanket (37.0 °C). A catheter (tubing: P10, Instech Solomon, USA; needle: BD Microlance™ 30G, BD, USA) was placed in the tail vein for later iv bolus administration of the tracer. Afterwards, the animals were positioned on the heated bed of the scanner. Parallel to the start of the 60-min dynamic µPET acquisition, a bolus of [^11^C]UCB-J was administered using an automated syringe pump at a rate of 1 mL/min (model 11 Elite, Harvard Apparatus, USA). Subsequently, a 10-min 80 kV/500 μA CT scan was acquired for attenuation and scatter correction. The acquisitions (60 + 10 min; frames: 12 × 10s, 3 × 20s, 3 × 30s, 3 × 60s, 3 × 150s, and 9 × 300s) were performed on two Siemens Inveon μPET/CT scanners (Siemens Preclinical Solutions, USA). During the scanning procedures, both the respiratory and the heart rate were monitored and the body temperature was kept at 37.0 °C with a feedback air flow system (Minerve, France). To account for the impact of anaesthesia, a wt animal and a ko animal were always scanned side-by-side receiving the same percentage of isoflurane from the evaporator. The animal and the scan parameters are represented in Table 1.

### Image processing

For quantitative image analysis, μPET images were reconstructed using a two-dimensional ordered subset expectation maximization (2D-OSEM) with four iterations and 16 subsets after Fourier rebinning. The images were reconstructed on a 128 × 128 × 159 grid with a voxel size of 0.776 × 0.776 × 0.796 mm. Normalization, dead time correction, random subtraction, CT-based attenuation correction, single-scatter simulation scatter corrections, and parallax corrections through detector response modelling were applied. Reconstructed images were processed in PMOD v3.6 (PMOD Technologies, Switzerland). A static image corresponding to the time-averaged frames of each dynamic acquisition was spatially transformed to a mouse brain [^11^C]UCB-J PET template (in house). This PET template already corresponded to a standardized MR template space (Waxholm MR) [49] with the corresponding volume of interest (VOI) definitions. The obtained matrix from the aforementioned brain normalization step was applied to transform all dynamic scans to the [^11^C]UCB-J template space. The regional time–activity curves were extracted from the resulting raw nonsmoothed images via the superimposition of the VOI template. The image-derived input function (IDIF) was obtained from the dynamic PET image via the extraction of the whole blood activity in the left ventricle of the heart using the CT image, as described previously [50]. The time-activity curves and the IDIF served as input for the Logan plot [51], resulting in the volume of distribution (*V*_T(IDIF)_), for [^11^C]UCB-J for the different brain regions of interest. The *V*_T(IDIF)_ consists of the specific (V_S_) and nondisplaceable (V_ND_) volume of distribution. This kinetic modelling method was recently shown to be suitable for reliable [^11^C]UCB-J *V*_T(IDIF)_ estimations in mice [31]. Additional pixelwise kinetic modelling was performed to generate parametric *V*_T(IDIF)_ images for each animal, again using the Logan plot. Subsequently averaged *V*_T(IDIF)_ images were generated for both groups at both timepoints. These images were smoothed using an isotropic Gaussian filter (FWHM = 0.5 mm), for visualization purposes.

### Ex vivo autoradiography

The gold standard to cross-validate [^11^C]UCB-J µPET measurements is [^11^C]UCB-J ex vivo autoradiography. A live animal is injected with the tracer prior to the sacrifice for tissue collection, in contrast to in vitro autoradiography (cfr. section "In vitro autoradiography") where the brain slides are incubated with the tracer. However, ex vivo autoradiography is hampered by the fast decay of ^11^C and by a relatively low injected dose in association with the imposed tracer dose conditions to mimic the µPET protocol. Here, we succeeded in injecting six animals with [^11^C]UCB-J and process their brain tissue simultaneously (*n* = 3/genotype) to enable the simultaneous exposure of the brain sections of all six animals to a single imaging plate for robustness.

A group of six anesthetized mice (*n* = 3/genotype; age 3 mo) received an iv bolus of [^11^C]UCB-J via the tail vein within a time frame of six minutes (injected dose wt: 3.29 ± 0.09, ko: 3.00 ± 0.09 MBq; cold mass wt: 4.57 ± 0.20, ko: 4.81 ± 0.11 nmol/kg). After an uptake period of 15 min, animals were killed, and brains were rapidly removed and snap frozen in 2-methylbutane (− 35 °C, 2 min). For each animal, nine serial sagittal brain sections (40 µm) were collected on Superfrost Plus slides (Thermo Fischer Scientific, USA) using a cryostat (Leica, Germany) with Paxinos and Watson coordinates (4th edition, 2013) as a reference (1.56 mm lateral). Based on the injected dose and an estimation of the percentage of tracer that reaches the brain from the µPET image, a range of tracer dilutions was prepared to obtain a standard curve (kBq per µL of reference solution, kBq/µL) representative for the amount of tracer present in the brain tissue. Forty-five minutes after the sacrifice, a phosphor imaging plate (BAS-IP MS2040 E, Fujifilm, Japan) was placed on the air-dried slides together with a range of standard dilutions (2 µL drops on Benchkote) in a light-impermeable cassette (Hypercassette, Amersham Biosciences, UK) for a total exposure of 90 min. Afterwards, the plate was scanned with a plate reader (Typhoon FLA7000, pixel size 25 µm, GE Healthcare, USA). The protocol was repeated for six 9-mo old animals (*n* = 3/genotype; injected dose wt: 4.59 ± 0.87, ko: 4.00 ± 0.10 MBq; cold mass wt: 4.49 ± 0.39, ko: 4.75 ± 0.07 nmol/kg).

### In vitro autoradiography

A second method to cross-validate the obtained [^11^C]UCB-J µPET results is [^3^H]UCB-J in vitro autoradiography. [^3^H]UCB-J (Novandi Chemistry AB, Sweden) was synthesized from tritium gas and purified via HPLC. The molar radioactivity of [^3^H]UCB-J was 81 Ci/mmol, and the radiochemical purity was > 99%. The tissue collection was identical as for in vivo autoradiography with a tissue thickness of 20 µm and additional storage at − 80 °C. The sections were thawed at room temperature, pre-incubated for 20 min with binding buffer (50 mM Tris–HCl buffer, pH 7.4), and dried using an airflow. Per animal (*n* = 5/genotype; 3-mo satellite animals and 9-mo animals randomly selected from the µPET cohorts), three sections were incubated with total binding (TB) solution (1 nM of [^3^H]UCB-J in binding buffer) and three sections with nonspecific binding (NB) solution (1 nM of [^3^H]UCB-J + 10 μM of cold UCB-J in binding buffer) for 60 min at room temperature. On ice, all sections were washed twice in 50 mM Tris–HCl buffer, followed by 5 dips in aq. The sections were air-dried at room temperature for 2 h to prepare for exposure on an imaging plate (BAS-IP TR2040 E, Fujifilm, Japan). All sections were exposed for a total duration of 72 h, together with a tritium standard for later quantification (American Radiolabeled Chemicals Inc., USA). Again, the plate reading was carried out using the Typhoon FLA7000 (GE Healthcare, USA) with a pixel size of 25 µm.

### Data and statistical analysis

All statistics were performed in GraphPad Prism 8 (GraphPad Software, USA). Concerning the µPET experiment, all animals with a missing observation at 9 mo were not included in the analysis (two wt animals). One wt animal died immediately after catheterization at the 3-mo timepoint. Cross-sectional VOI-based [^11^C]UCB-J µPET data were analyzed using a two-way ANOVA with post hoc Bonferroni correction for multiple comparisons, thereby comparing the genotypes at both timepoints separately. A mixed-effect model was used for the analysis of repeated-measures data, thereby allowing the two missing observations in the wt group. The model was fitted with genotype (wt and ko) x time (3 mo and 9 mo) as fixed effects and subject as a random effect. An additional post hoc Bonferroni multiple comparison test was performed. All µPET data are expressed as the averaged *V*_T(IDIF)_ ± standard deviation (SD). Also, a voxel-based analysis was performed on the smoothed the *V*_T(IDIF)_ images using Statistical Parametric Mapping (SPM) 12 (Wellcome Department of Imaging Neuroscience, UK) in MATLAB (R2016a, The Mathworks Inc, USA). Statistical T-maps were calculated at a significance level of *p =* 0.01 and a cluster threshold of 100 voxels (0.8 mm^3^).

For both autoradiography methods, the obtained digital image was processed using ImageJ software (National Institutes of Health, USA). The different regions of interest were manually delineated to extract mean gray values. Subsequently, these values were interpolated on the corresponding standard curve to obtain the [^11^C]UCB-J tissue concentration (kBq/mL) for ex vivo autoradiography and [^3^H]UCB-J total binding (TB) (nCi/mg) for in vitro autoradiography. For ex vivo autoradiography, nine sections were analyzed for each genotype to obtain regional (cortex, striatum, thalamus, and hippocampus) [^11^C]UCB-J tissue concentrations (kBq/mL) consisting of both the specific and nonspecific binding of the radioligand. These values were decay corrected to the start of the exposure and based on these decay corrected values the standard uptake value (SUV) was calculated (activity concentration/ [injected dose/body weight]).

The in vitro autoradiography quantification resulted in regional (cortex, striatum, thalamus, and hippocampus) values for total binding (TB) and nonspecific binding (NB). Based on these parameters, [^3^H]UCB-J specific binding (SB) (nCil/mg) was calculated (SB = TB–NB). Three sections per animal were analyzed. The regional averaged SUV and SB (± SD) values were compared between genotypes and timepoints using unpaired t tests.

Pearson’s r correlation was used to determine the relationship of the averaged regional [^11^C]UCB-J µPET measurements versus both the [^11^C]UCB-J ex vivo and the [^3^H]UCB-J in vitro autoradiography data.

## Results

### Lower synaptic density in the brain of young adult Sapap3 ko mice.

**Fig. 2:**
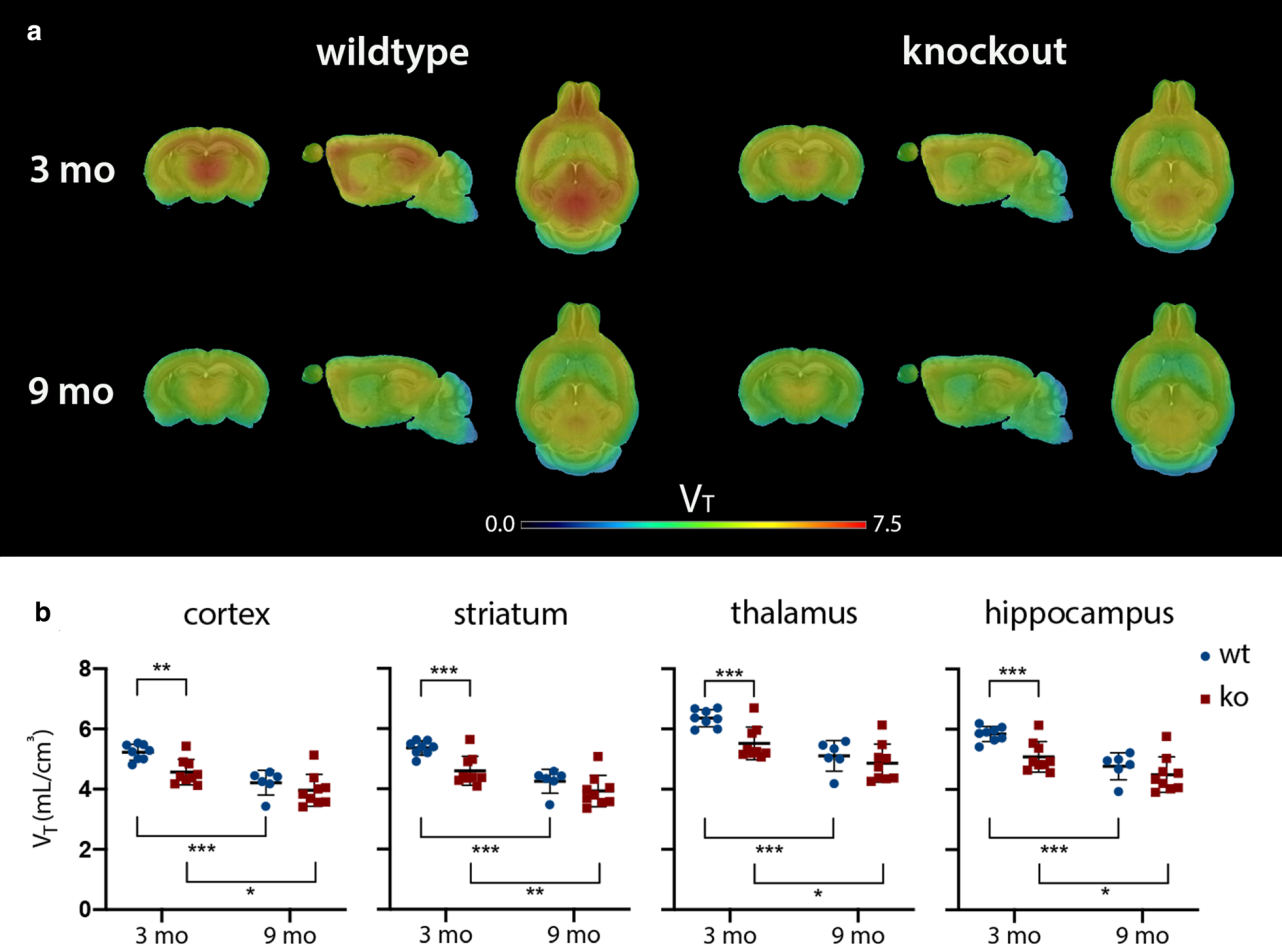
**a** The averaged [^11^C]UCB-J PET *V*_T(IDIF)_ maps superimposed on a mouse MR template at 3 and 9 mo for both genotypes, **b** combined with the corresponding averaged [^11^C]UCB-J PET *V*_T(IDIF)_ values ± SD. Young adult Sapap3 ko mice are characterized by a lower synaptic density availability in the cortex, the striatum, the thalamus, and the hippocampus when compared to their wt counterparts. Also, both genotypes show a progressive decline in synaptic density availability with ageing, which was more pronounced in the wt controls. The corresponding averaged values ± SD can be consulted in Additional file 1: Table 1. (ko, knockout; mo, months; MR, magnetic resonance; wt, wildtype; *V*_T(IDIF)_, volume-of-distribution; *p < 0.05, **p < 0.01, ***p < 0.001)

A cross-sectional comparison in young adult mice (3 mo) revealed significantly lower *V*_T(IDIF)_ values for the ko group in the cortex (− 12.69 ± 3.31%; *p =* 0.0045), the striatum (− 14.12 ± 3.53%; *p =* 0.0010), the thalamus (− 13.11 ± 3.37%; *p =* 0.0003), and the hippocampus (− 12.99 ± 3.38%; *p =* 0.0009) compared to the wt control group. At 9 mo, the same comparison did not reach significance for any of the aforementioned regions (Fig. 2).

Both the wt and the ko mice showed a significant age-dependent decline in *V*_T(IDIF)_ (Fig. 2). For wt animals, this significant decrease (*p <* 0.001) was present in all regions studied: the cortex (− 19.42 ± 4.35%), the striatum (− 20.49 ± 4.36%), the thalamus (− 19.73 ± 4.35%), and the hippocampus (− 18.41 ± 4.37%). In ko mice, this significant *V*_T(IDIF)_ decline was less pronounced: the cortex (− 13.25 ± 4.35%), the striatum (− 14.62 ± 4.43%), the thalamus (− 11.96 ± 4.37%), and the hippocampus (− 11.60 ± 4.39%).

**Fig. 3:**
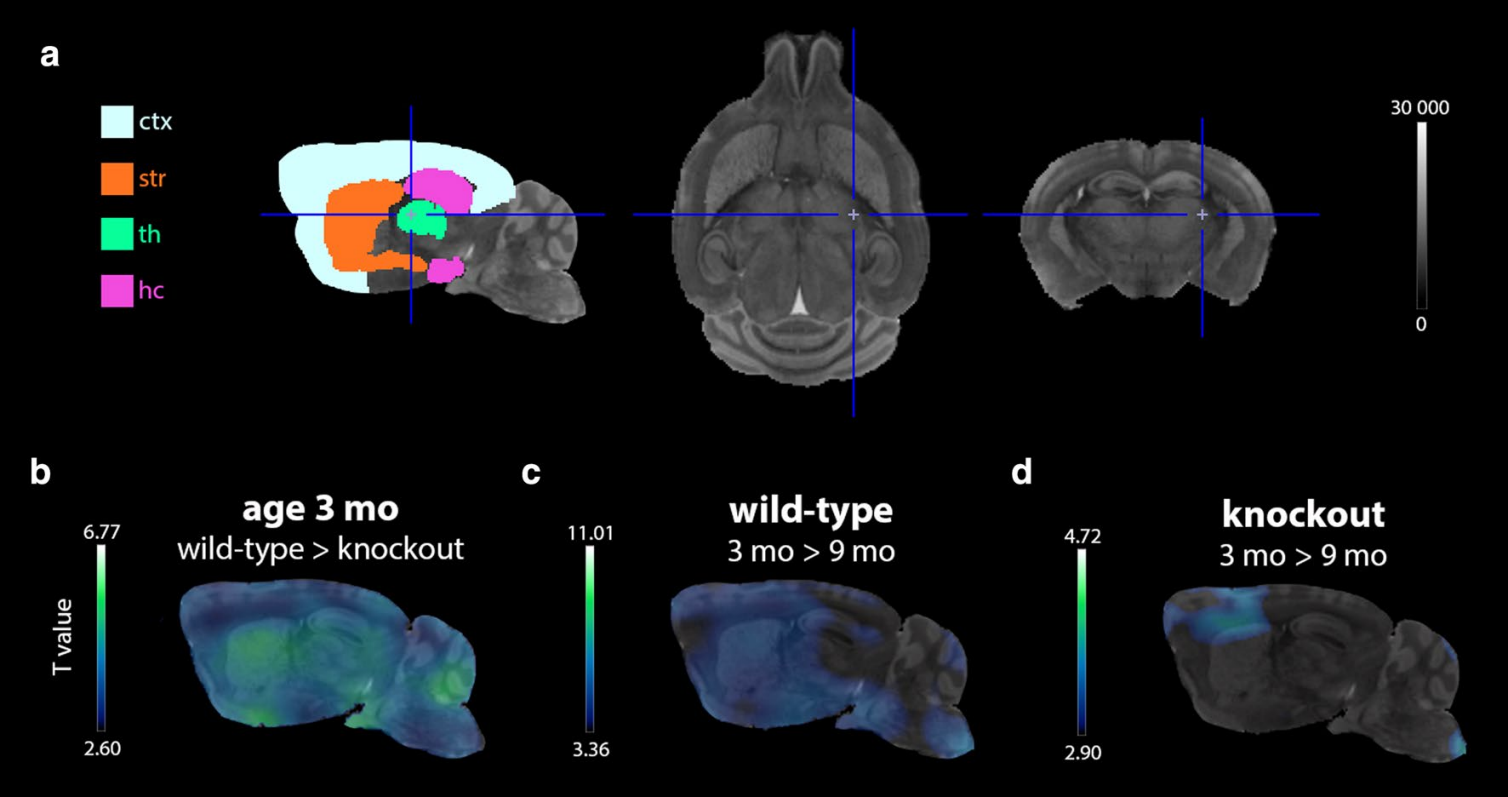
The results of the voxel-based statistical parametric mapping analysis of the [^11^C]UCB-J *V*_T(IDIF)_ maps. **a** A representation of all analysed brain regions on a corresponding MR template. **b** Hypo T-map showing all voxel clusters (threshold: 100 voxels) with a significantly (*p <* 0.01) lower *V*_T(IDIF)_ in ko compared to wt mice at the age of 3 mo. **c** Hypo T-maps indicating all voxel clusters (threshold: 100 voxels) that significantly (*p <* 0.01) declined over time in wt mice. **d** Similar hypo T-maps as in panel **c** for the ko mice (ctx = cortex; hc = hippocampus; ko = knockout; mo = months; MR = magnetic resonance; str = striatum; th = thalamus; wt = wildtype)

The additional SPM voxel-based analysis confirmed the results of the VOI-based analysis. Figure 3 displays the resulting T-maps (b–d) indicating the voxels with a significantly lower *V*_T(IDIF)_ (*p <* 0.01; cluster threshold = 100 voxels) for the listed comparisons. For all studied regions, a cross-sectional comparison at 3 mo revealed a substantial % of voxels with a significantly (*p <* 0.01) lower *V*_T(IDIF)_ in ko animals (Fig. 3b) within the cortex (88.69%), the striatum (99.86%), the thalamus (100.00%), and the hippocampus (96.95%). At 9 mo, no voxels clusters with a significantly lower *V*_T(IDIF)_ were found at *p <* 0.01. Longitudinal analysis identified all voxels with a significantly (*p <* 0.01) lower *V*_T(IDIF)_ at age 9 mo for both the wt (all studied regions: cortex (85.70%), striatum (99.99%), thalamus (99.05%), hippocampus (85.35%); Fig. 3c) and ko (cortex (16.99%) and striatum (19.70%); Fig. 3d) group.

### [^11^C]UCB-J ex vivo autoradiography as a proxy for in vivo [^11^C]UCB-J µPET

**Fig. 4:**
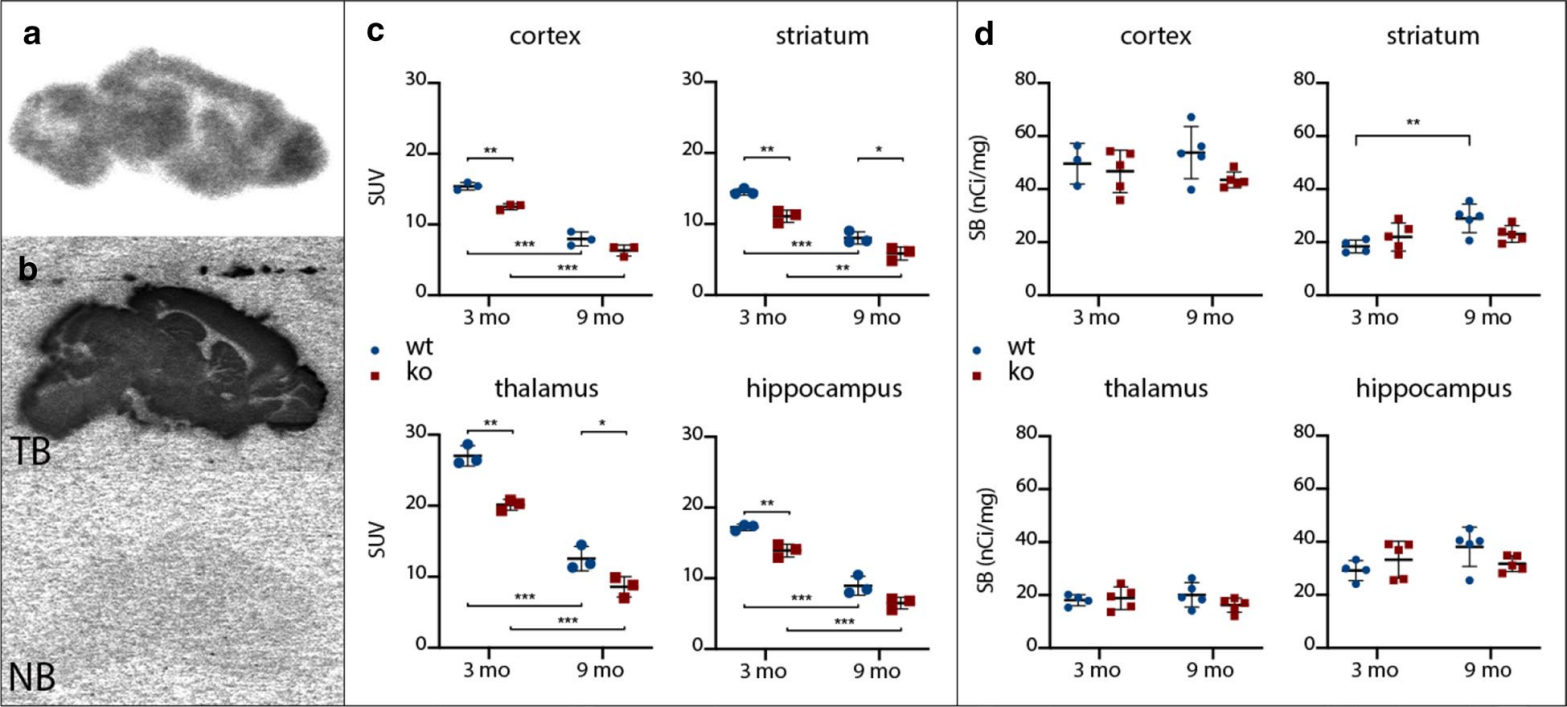
Representative sagittal autoradiograms of **a** [^11^C]UCB-J ex vivo and **b** [^3^H]UCB-J in vitro autoradiography with the corresponding regional **c** standard uptake values (SUV) of [^11^C]UCB-J ex vivo autoradiography and **d** specific binding (SB) values of [^3^H]UCB-J in vitro autoradiography at the age of 3 mo and 9 mo. The corresponding averaged values ± SD can be consulted in Additional file 1: Table 2. (ko = knockout; mo = months; NB = nonspecific binding; TB = total binding; wt = wildtype; *p < 0.05; **p < 0.01; ***p < 0.001)

**Fig. 5:**
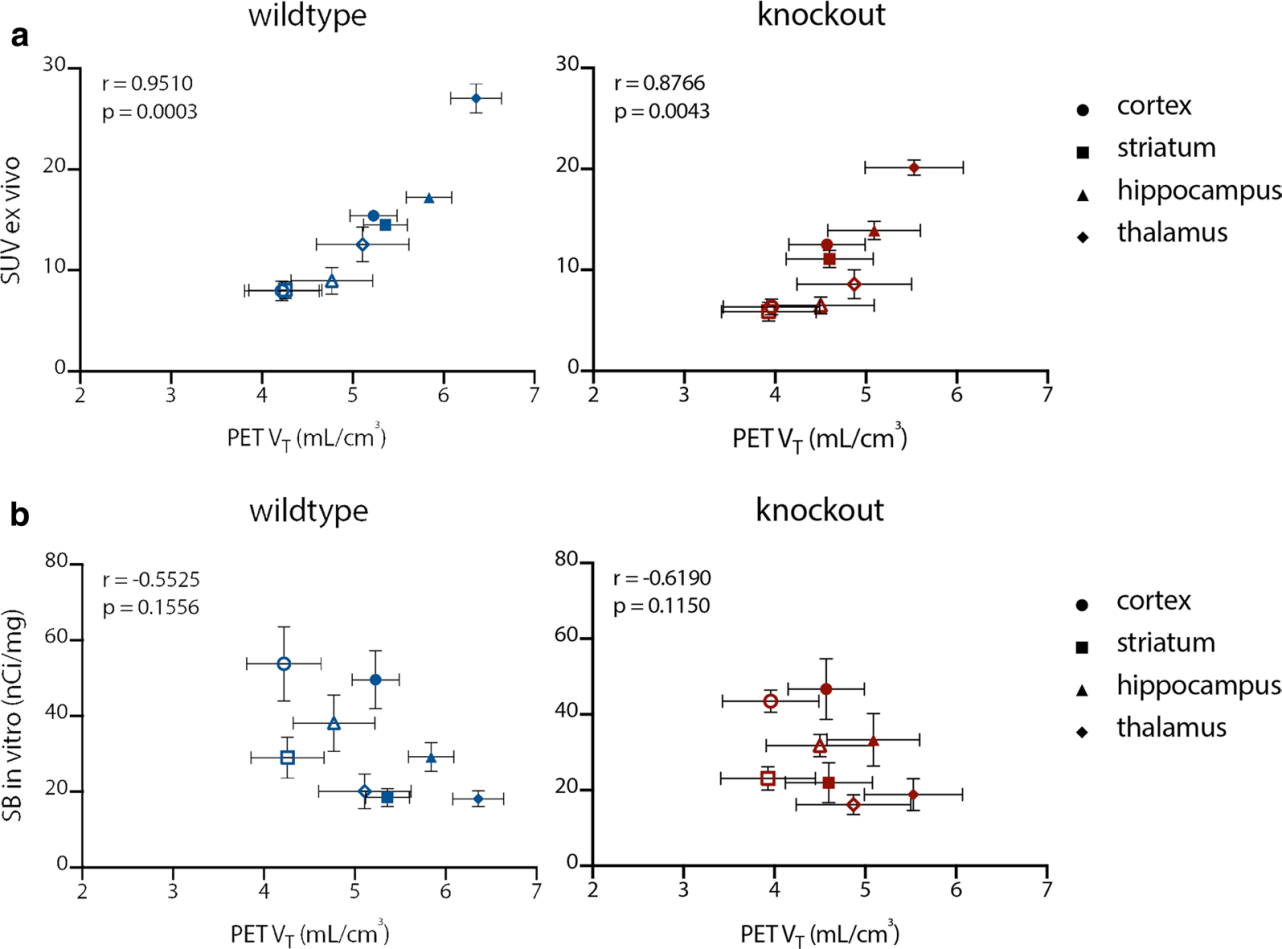
The averaged [^11^C]UCB-J µPET volume of distribution (*V*_T(IDIF)_) ± SD **a** versus the [^11^C]UCB-J ex vivo autoradiography averaged standard uptake value (SUV) ± SD and **b** versus the [^3^H]UCB-J in vitro autoradiography averaged specific binding (SB) for both genotypes. Three-mo data are depicted as filled symbols, while 9-mo data are depicted as open symbols

A comparison of [^11^C]UCB-J ex vivo autoradiography results between genotypes revealed a significantly (*p <* 0.01) lower SUV in ko mice at the age of 3 mo for the cortex (− 18.75 ± 2.53%; *p =* 0.0017), the striatum (− 23.55 ± 3.83%; *p =* 0.0035), the thalamus (− 25.49 ± 3.45%; *p =* 0.0018), and the hippocampus (− 19.12 ± 3.40%; *p =* 0.0049) (Fig. 4c). At 9 mo, the same comparison only showed a significantly (*p <* 0.05) lower SUV in the striatum (− 27.10 ± 9.00%; *p =* 0.0395) and the thalamus (− 31.63 ± 10.29%; *p =* 0.0371) of ko mice compared to wt controls (Fig. 4c). When comparing the means of the wt control group between both ages, all regions studied [the cortex (− 48.33 ± 4.17%), the striatum (− 44.58 ± 3.81%), the thalamus (− 53.57 ± 4.76%), and the hippocampus (− 47.94 ± 4.71%)] showed a significantly (*p <* 0.001) lower SUV at the 9-mo timepoint. This also applies for the ko animals at the two different ages [the cortex (− 106.07 ± 6.40%; *p <* 0.001), the striatum (− 105.54 ± 9.02%; *p =* 0.0020), the thalamus (− 104.27 ± 7.49%; *p <* 0.001), and the hippocampus (− 105.35 ± 7.97%; *p =* 0.0020)]. Also, the regional averaged [^11^C]UCB-J ex vivo autoradiography and the [^11^C]UCB-J µPET *V*_T(IDIF)_ measurements were (Fig. 5a) shown to be significantly correlated for both genotypes (wt: *r* = 0.9510, *p <* 0.001; ko: *r* = 0.8766, *p <* 0.01).

Concerning the [^3^H]UCB-J in vitro autoradiography data, no significant differences were found between genotypes or timepoints, except for a significantly higher specific binding (SB) in 9-mo-old wt animals compared to those at the age of 3 mo in the striatum (+ 57.50 ± 15.99; *p =* 0.0088) (Fig. 4d). No significant correlation was found between the [^3^H]UCB-J in vitro autoradiography SB and the averaged regional [^11^C]UCB-J µPET *V*_T(IDIF)_ (Fig. 5b).

## Discussion

In the present study, we aimed to describe cross-sectional differences in the synaptic density between wt controls and Sapap3 ko mice, which progress to severe compulsive-like grooming behaviour [27], thereby using the newly established PET marker [^11^C]UCB-J. Also, we studied longitudinal changes in this marker during grooming aggravation, for each genotype separately. To date, this is the first study to verify whether [^11^C]UCB-J ex vivo autoradiography is suitable to validate [^11^C]UCB-J µPET results.

This study demonstrated a significantly lower in vivo SV2A availability in young adult Sapap3 ko mice (3 mo), both in- and outside of the CSTC “OCD” brain circuit. A possible explanation includes a difference in synaptic pruning between genotypes, as this process has already finished at the age of 3 mo in mice [52]. Indeed, abnormal synaptic pruning was observed in progranulin-deficient mice which also develop excessive grooming [53]. Synaptic pruning might also be altered in mice intranasally infected with Group A Streptococcus, which function as a model for PANDAS (Paediatric Autoimmune Neuropsychiatric Disorders Associated with Streptococcal Infections, characterized by sudden onset OCD) [54, 55]. Moreover, the offspring of pregnant Sapap3 ko mice injected with the non-nucleoside DNA methyltransferase inhibitor RG108, which affects synaptic pruning, showed a 4-month delay in the development of repetitive grooming behaviour [56], whereas a similar injection in adult Sapap3 ko mice ameliorated such behaviour for only 3 days [57]. Parallel, a series of clinical imaging studies suggest abnormal synaptic pruning patterns in, among others, frontostriatal brain structures as a possible explanation for volumetric abnormalities in paediatric OCD patients [58, 59]. On the other hand, the lower [^11^C]UCB-J availability in 3-mo old Sapap3 ko mice could also be explained by deviations in synaptogenesis secondary to disruption of the PSD-95/Sapap/Shank complex, with its pivotal role in synaptic development and function [21]. Like Sapap3 ko mice, both PSD-95 ko mice and multiple Shank mutant mouse models show excessive self-grooming and anxiety-like behaviour [51, 52]. Previously, PSD-95/Sapap disruption was shown to impair synaptic maturation and to weaken synaptic strength [21]. Likewise, Shank is involved in the morphological and functional maturation of synapses [62]. Furthermore, Sapap3 is connected via PSD-95 to a transsynaptic cell-adhesion molecule involved in presynaptic differentiation, i.e. neuroligin [63, 64]. Mice lacking neuroligin1 also show excessive grooming behaviour [65]. A study in Sapap3 ko mice by Chen and colleagues identified changes in presynaptic function with excessive synaptic depression of medium spiny neuron excitatory synapses mediated via retrograde endocannabinoid signalling [23]. So, disturbances in synaptic pruning, synaptogenesis, and/or abnormalities in synaptic protein complexes could provide possible explanations for why in vivo loss of a postsynaptic protein (i.e. Sapap3) alters the availability of a presynaptic protein (i.e. SV2A) for [^11^C]UCB-J.

Interesting, Welch and colleagues [11] already investigated synaptic alterations in Sapap3 ko mice in vitro. They found no difference in presynaptic function and spine density on striatal medium spiny neurons in Sapap3 ko mice. Also, PSD-95 and Shank1-3 levels from striatal PSD fractions were similar for adult ko and wt mice. However, they were able to demonstrate the presence of immature synapses in ko mice which may be related to our findings as we show that SV2A availability evolves differently with age when comparing ko mice with wt controls.

Secondly, despite an already very low baseline SV2A availability, Sapap3 ko mice still showed a significant further and more focal decline in synaptic density when progressing to a more severe phenotype. Based on the voxel-based SPM results this was more pronounced at the corticostriatal level, previously shown to be affected in this model [11, 17, 22–26] and to be relevant to OCD [66], whereas healthy aging in wt mice was associated with a more pronounced and diffuse significant decline in SV2A availability throughout the brain. Besides postnatal data [67, 68], there are currently no data available on the evolution of SV2A levels in the healthy rodent brain during adult life. Human [^11^C]UCB-J PET studies already showed an age-related decline in synaptic density in healthy controls [69, 70]. Based on this diffuse decline in the brain of wt controls, genotypes no longer showed a cross-sectional difference at the age of 9 mo based on both the VOI-(*p <* 0.05) and the voxel-based (*p <* 0.01) analysis. Yet when imposing a significance level of *p <* 0.05 for the voxel-based analysis, 16.89% of all voxels within the total striatal volume of ko mice had a significantly lower *V*_T(IDIF)_, relative to the wt group. This supports the presence of a more focal decline, limited to the striatum, in Sapap3 ko mice.

In general, PET experiments are limited by spatial resolution leading to partial-volume effects that may affect quantification. Therefore, in vivo PET data were cross-validated using two types of autoradiography. Compared to [^3^H]UCB-J in vitro autoradiography, where brain slides are directly incubated with the tracer, [^11^C]UCB-J ex vivo autoradiography is characterized by the injection of live animals with the tracer and is technically more challenging. This ex vivo autoradiography protocol requires a simultaneous processing of the brain tissue of multiple animals injected with a low tracer dose of the radioligand [47], which requires a sub-hour protocol given the fast decay of the ^11^C isotope (20.39 min). However, when these practical limitations are overcome, the ex vivo approach is the gold standard for the validation of µPET data due to its superior ability to mimic what happens in vivo. Indeed, a strong linear relationship was established between the outcome parameters of [^11^C]UCB-J µPET and [^11^C]UCB-J ex vivo autoradiography. Moreover, at the 9-mo timepoint, ex vivo autoradiography was able to pick up significant differences between the wt and ko group, which did not emerge from the µPET data. This could be based on its higher spatial resolution and hence less associated partial-volume effects. Also, a brain region may be characterized by a heterogeneous distribution of the target. When quantifying µPET data, the binding of [^11^C]UCB-J in the entire brain region is considered, whereas with classic autoradiography the binding of the radioligand is only measured in a fraction of the region. However, with autoradiography, each animal can only be studied once which could be also an extra source of variability. Furthermore, no relationship could be established between [^11^C]UCB-J PET and [^3^H]UCB-J in vitro autoradiography outcome parameters. The thalamus, for example, behaves differently in vitro versus in vivo. For in vivo PET and ex vivo autoradiography, the binding outcome parameter for the thalamus is relatively high compared to the other three analysed regions, while in vitro, the thalamus is characterized by relatively lower values. These findings could be explained by methodological differences between in vivo (PET and autoradiography) and in vitro (autoradiography) protocols. For both [^11^C]UCB-J PET and ex vivo autoradiography, the tracer [^11^C]UCB-J is physiologically perfused and distributed throughout the tissue, whereas with [^3^H]UCB-J in vitro autoradiography, the section is directly incubated with the ligand. Recently, Thomsen and colleagues validated [^11^C]UCB-J µPET results using [^3^H]UCB-J in vitro autoradiography in both the quinolinic acid rat model for Huntington’s disease and in the Göttingen minipig [71, 72]. In the rat study [71], unilateral striatal loss of SV2A binding ([^11^C]UCB-J µPET) ipsilateral to the injection site of quinolinic acid was confirmed in coronal brain sections using [^3^H]UCB-J in vitro autoradiography. Since this model is characterized by striatal cell loss at the level of the injection site [73], an impact on the binding of both [^11^C]UCB-J and [^3^H]UCB-J is likely. In contrast to the study in rats, the minipig study reported a significant correlation between [^11^C]UCB-J PET *V*_T(IDIF)_ and [^3^H]UBC-J in vitro autoradiography specific binding for multiple brain regions. Unlike in this study, a white matter region (centrum semiovale) was included in this correlation. They show that upon exclusion of this region, the correlation was lost, thereby supporting our finding of a lack of correlation (Fig. 5b). Moreover, these studies concern different species.

Although the clinical application of [^11^C]UCB-J PET is promising, to date, no clinical PET studies investigated SV2A availability in OCD patients. As the evidence for synaptic abnormalities in neuropsychiatric disorders, i.a. OCD, is emerging [32–36], further evaluation of SV2A availability in patients could be of importance. This also applies to other neuropsychiatric disorders known to be associated with synaptic alterations and/or mutations in genes encoding for PSD scaffolding proteins, i.e. autism spectrum disorder, schizophrenia, intellectual disability, etc. [38–40]. Here, we show secondary presynaptic changes in SV2A availability upon postsynaptic Sapap3 loss. However, a possible role for SV2A itself driving compulsions was previously demonstrated in a clinical setting. Epilepsy patients were shown to develop compulsive behaviours upon administration of the SV2A-targeting antiepileptic drug levetiracetam [42, 44, 45, 74].

More work will be necessary to fully understand whether changes in SV2A availability for [^11^C]UCB-J imply a decreased SV2A density in vesicles, dysfunctional SV2A, a decreased number of vesicles or synapses, or neuronal loss [75]. Moreover, this may also differ depending on the studied disease or model. Currently, a good correlation was already established between the histopathological synaptic marker synaptophysin and [^11^C]UCB-J binding in baboons [28]. On the other hand, a study in mice suggests that hippocampal SV2A loss does not alter synaptic density or morphology [76]. Therefore, one should carefully interpret [^11^C]UCB-J PET data and ideally perform a cross-validation of results using autoradiography or other ex vivo techniques, where possible.

In conclusion, we established that young adult Sapap3 ko mice start off a lower in vivo SV2A availability for [^11^C]UCB-J. Upon aggravation of excessive self-grooming, this level further declines in brain regions with relevance to OCD. With this study, we show the potential of the synaptic density marker [^11^C]UCB-J PET to function as a predictor and hence biomarker for the development of compulsive-like grooming. In addition, we suggest [^11^C]UCB-J ex vivo autoradiography, as a suitable proxy for [^11^C]UCB-J PET data in mice.

## Supplementary information

**Additional file 1: Supplementary Table 1**. Overview of the averaged [^11^C]UCB-J µPET* V*_T(IDIF)_ values ± SD of the wildtype versus the knockout mice at both timepoints with the corresponding % cross-sectional difference ± SE (mo = months; ko = knockouts;* V*_T(IDIF)_ = volume of distribution; wt = wildtypes; **p<0.01; ***p<0.001). **Supplementary Table 2**. Overview of the averaged [^11^C]UCB-J ex vivo autoradiography standard uptake values (SUV) ± SD and [^3^H]UCB-J in vitro autoradiography specific binding (SB) ± SD of the wildtype versus the knockout mice at both timepoints with the corresponding % cross-sectional difference ± SE (mo = months; ko = knockouts; wt = wildtypes; *p<0.05; **p<0.01).

## Data Availability

The data that support the findings of this study are available from the corresponding author upon reasonable request.

## Abbreviations

[^11^C]UCB-J: (R)-1-((3-((11)C-methyl-(11)C)pyridin-4-yl)methyl)-4-(3,4,5-trifluorophenyl)pyrrolidin-2-one
CSTC: Cortico-striato-thalamocortical
IDIF: Image-derived input function
ko: Knockout
mo: Months
NB: Nonspecific binding
OCD: Obsessive–compulsive disorder
PSD: Postsynaptic density
Sapap3; DLGAP3; GKAP3: SAP90/PSD-95-associated protein
SB: Specific binding
SPM: Statistical parametric mapping
SUV: Standard uptake value
SV2A: Synaptic vesicle protein 2A
TB: Total binding
VOI: Volume of interest
*V*_ND_: Nondisplaceable volume of distribution
*V*_S_: Specific volume of distribution
*V*_T__(IDIF)_: Volume of distribution
wt: Wildtype
µPET: Small-animal positron emission tomography
