# Supplementary material for: Sapap3 deletion causes dynamic synaptic density abnormalities: a longitudinal [11C]UCB-J PET study in a model of obsessive–compulsive disorder-like behaviour
Source: EJNMMI Res. 2020 Nov 13;10:140. doi: 10.1186/s13550-020-00721-2 (PMC7666267; doi:10.1186/s13550-020-00721-2)
Supplement: Supplementary file 1 — Additional file 1: Supplementary Table 1. Overview of the averaged [11C]UCB-J µPET VT(IDIF) values ± SD of the wildtype versus the knockout mice at both timepoints with the corresponding % cross-sectional difference ± SE (mo = months; ko = knockouts; VT(IDIF) = volume of distribution; wt = wildtypes; **p<0.01; ***p<0.001). Supplementary Table 2. Overview of the averaged [11C]UCB-J ex vivo autoradiography standard uptake values (SUV) ± SD and [3H]UCB-J in vitro autoradiography specific binding (SB) ± SD of the wildtype versus the knockout mice at both timepoints with the corresponding % cross-sectional difference ± SE (mo = months; ko = knockouts; wt = wildtypes; *p<0.05; **p<0.01). [file 13550_2020_721_MOESM1_ESM.docx]

**SUPPLEMENTARY MATERIAL**

| **Age** | **Region** | **wt**  **V_T (IDIF)_** | **ko**  **V_T (IDIF)_** | **Cross-sectional difference (%)** |
| --- | --- | --- | --- | --- |
| **3 mo** | Cortex | 5.23 ± 0.26 | 4.57 ± 0.42 | -12.69 ± 3.31** |
|  | Striatum | 5.36 ± 0.23 | 4.60 ± 0.48 | -14.12 ± 3.53*** |
|  | Thalamus | 6.36 ± 0.28 | 5.53 ± 0.54 | -13.11 ± 3.37 *** |
|  | Hippocampus | 5.84 ± 0.25 | 5.09 ± 0.51 | - 12.99 ± 3.38 *** |
| **9 mo** | Cortex | 4.22 ± 0.41 | 3.96 ± 0.53 | -6.00 ± 6.09 |
|  | Striatum | 4.26 ± 0.40 | 3.93 ± 0.52 | - 7.66 ± 5.91 |
|  | Thalamus | 5.11 ± 0.51 | 4.87 ± 0.63 | -4.70 ± 6.04 |
|  | Hippocampus | 4.77 ± 0.45 | 4.50 ± 0.59 | -5.73 ± 5.97 |

**SUPPLEMENTARY TABLE 1.** Overview of the averaged [^11^C]UCB-J µPET V_T (IDIF)_ values ± SD of the wildtype versus the knockout mice at both timepoints with the corresponding % cross-sectional difference ± SE (mo = months; ko = knockouts; V_T (IDIF)_ = volume of distribution; wt = wildtypes; **p<0.01; ***p<0.001)

|  |  | *[^11^C]UCB- J ex vivo autoradiography* | | | *[^3^H]UCB- J in vitro autoradiography* | | |
| --- | --- | --- | --- | --- | --- | --- | --- |
| **Age** | **Region** | **wt**  **SUV** | **ko**  **SUV** | **Cross-sectional difference (%)** | **wt**  **SB**  **(nCi/mg)** | **ko**  **SB**  **(nCi/mg)** | **Cross-sectional difference (%)** |
| **3 mo** | Cortex | 15.41 ± 0.53 | 12.52 ± 0.42 | -18.75 ± 2.52** | 49.58 ± 7.70 | 46.73 ± 8.01 | -5.75 ± 11.65 |
|  | Striatum | 14.52 ± 0.43 | 11.10 ± 0.86 | -23.55 ± 3.83** | 18.40 ± 2.43 | 21.96 ± 5.30 | +19.35 ± 15.72 |
|  | Thalamus | 27.03 ± 1.42 | 20.14 ± 0.76 | -25.49 ± 3.45** | 18.07 ± 2.07 | 18.81 ± 4.24 | +4.10 ± 12.92 |
|  | Hippocampus | 17.21 ± 0.44 | 13.92 ± 0.91 | - 19.12 ± 3.40** | 29.17 ± 3.78 | 33.28 ± 6.93 | +14.09 ± 13.32 |
| **9 mo** | Cortex | 7.96 ± 0.98 | 6.34 ± 0.78 | -20.42 ± 9.08 | 53.77 ± 9.78 | 43.52 ± 2.99 | -19.06 ± 8.52 |
|  | Striatum | 8.05 ± 0.86 | 5.87 ± 0.92 | - 27.10 ± 9.00* | 28.98 ± 5.41 | 23.08 ± 3.11 | -20.36 ± 9.62 |
|  | Thalamus | 12.55 ± 1.71 | 8.58 ± 1.44 | -31.63 ± 10.29* | 20.08 ± 4.63 | 16.19 ± 2.63 | -19.37 ± 11.85 |
|  | Hippocampus | 8.96 ± 1.33 | 6.49 ± 0.83 | -27.56 ± 10.14 | 38.11 ± 7.40 | 31.74 ± 2.96 | -16.71 ± 9.35 |

**SUPPLEMENTARY TABLE 2.** Overview of the averaged [^11^C]UCB-J ex vivo autoradiography standard uptake values (SUV) ± SD and [^3^H]UCB-J in vitro autoradiography specific binding (SB) ± SD of the wildtype versus the knockout mice at both timepoints with the corresponding % cross-sectional difference ± SE (mo = months; ko = knockouts; wt = wildtypes; *p<0.05; **p<0.01)
